# Basic Timing Abilities Stay Intact in Patients with Musician's Dystonia

**DOI:** 10.1371/journal.pone.0092906

**Published:** 2014-03-25

**Authors:** M. C. van der Steen, Floris T. van Vugt, Peter E. Keller, Eckart Altenmüller

**Affiliations:** 1 Max Planck Institute for Human Cognitive and Brain Sciences, Research Group “Music Cognition and Action”, Leipzig, Germany; 2 Institute of Music Physiology and Musicians' Medicine, University of Music, Drama and Media, Hanover, Germany; 3 Lyon Neuroscience Research Center CNRS-UMR 5292, INSERM U1028, University Lyon-1, Lyon, France; 4 The MARCS Institute, University of Western Sydney, Sydney, Australia; IIT - Italian Institute of Technology, Italy

## Abstract

Task-specific focal dystonia is a movement disorder that is characterized by the loss of voluntary motor control in extensively trained movements. Musician's dystonia is a type of task-specific dystonia that is elicited in professional musicians during instrumental playing. The disorder has been associated with deficits in timing. In order to test the hypothesis that basic timing abilities are affected by musician's dystonia, we investigated a group of patients (N = 15) and a matched control group (N = 15) on a battery of sensory and sensorimotor synchronization tasks. Results did not show any deficits in auditory-motor processing for patients relative to controls. Both groups benefited from a pacing sequence that adapted to their timing (in a sensorimotor synchronization task at a stable tempo). In a purely perceptual task, both groups were able to detect a misaligned metronome when it was late rather than early relative to a musical beat. Overall, the results suggest that basic timing abilities stay intact in patients with musician's dystonia. This supports the idea that musician's dystonia is a highly task-specific movement disorder in which patients are mostly impaired in tasks closely related to the demands of actually playing their instrument.

## Introduction

Task-specific focal dystonia is a movement disorder that manifests itself as a loss of voluntary motor control in extensively trained movements [1]–[4]. Although this type of dystonia clearly impairs patients' ability to perform certain movements, it has been suggested that movement processing, planning, somatosensory functions and aspects of timing are also affected [5]–[6]. A well-known example of task-specific focal hand dystonia is writer's cramp. When picking up a pen or writing some words, dystonic postures of the hand occur that disrupt the speed and accuracy of writing [7]. Another form of task-specific dystonia is musician's dystonia (MD), which is characterized by impairments related to instrumental playing in professional musicians. With an estimated one percent of professional musicians being affected by MD, the prevalence of MD is much higher compared to other forms of focal dystonia in the general population [1]. This article focusses on the form of MD that affects the fingers and/or hand, leaving aside embouchure dystonia that affect the coordination of lips, tongue, facial and cervical muscles of brass and wind players. Typically the cramping, co-contractions of antagonist muscle groups that accompany the loss of motor control, as well as the dystonic postures during instrumental playing, occur without pain (although muscle aching can occur after lasting spasms) [1], [8]. In piano playing MD disrupts the fluidity of movements related to instrumental playing [9]. Furthermore, MD affects individuated finger movements, as evidenced by more forceful keystrokes and abnormal temporal control of the keystrokes [10]. For the affected musicians the disorder is very disabling and often signifies the end of a musical career [8]. Furthermore, as time passes, MD patients may show overflow of impairments to other tasks, such as hand writing or typing on a keyboard [11]–[13]. Several therapies are available to MD patients such as botulinum toxin injections [14] and behavioural retraining [13]. These therapies seem to have positive effect in about half of the patients but, unfortunately, the disorder often forces musicians to change profession [14].

The pathophysiology of MD is still unclear but both functional and structural abnormalities (i.e., maladaptive plasticity) in motor-related cortical and subcortical regions (e.g., primary motor cortex, supplementary motor area, basal ganglia and cerebellum) have been linked with focal hand dystonia [15]–[18]. For example, blurred or even overlapping somatosensory representations of the single fingers have been found in MD-patients [1]. Functional abnormalities have been shown both in relation to task-specific movements (guitar playing, writing) [19]–[20] and more general tasks, like finger tapping [21]. Furthermore, focal hand dystonia (MD and writer's cramp) patients showed reduced central nervous surround inhibition in the finger muscles when investigated with motor cortex stimulation [22]–[23]. In addition to these altered inhibition patterns at different levels of the central nervous system, alterations in sensorimotor integration play a role in focal hand dystonia [24]–[26].

The brain areas that show abnormalities in MD patients have previously been shown to be critical for different aspects of timing. Extensive networks of brain regions have been linked with sensorimotor synchronization, temporal processing, and the evaluation of temporal structures. Brain areas typically implicated with these types of timing behavior are the primary sensorimotor cortices, the inferior parietal cortex, supplementary motor area, the cerebellum, and the basal ganglia [27]–[28]. Problems due to disorders (e.g. Parkinson's disease) and lesions in these areas (e.g., the basal ganglia) have shown to compromise timing behavior [29]–[30]. The overlap in brain areas that show abnormalities in focal hand dystonia patients and the brain areas involved in sensorimotor synchronization-timing tasks made us hypothesize that MD patients might show impaired timing abilities.

Indeed, some previous studies found that focal hand dystonia patients have impaired perceptual timing and temporal processing abilities. For example, Lim and colleagues [3] had healthy controls, writer's cramp and MD patients, away from their instrument, judge whether a sequence of six brief pulses (auditory and tactile stimuli) appeared to be regular or not. The interval between the fifth and sixth pulse varied, creating regular and irregular sequences. Results showed that compared to controls, MD patients were less sensitive to these timing irregularities, both in the tactile and auditory domain. The writer's cramp patients did not show this impairment. A large study investigated somatosensory temporal discrimination in patients with various forms of focal dystonia by means of paired stimuli with an increasing inter-stimulus interval to the skin on different body parts. Like other groups of patients with focal dystonia, the patients with writer's cramp showed higher discrimination thresholds compared to healthy control subjects [31]. Further abnormalities of tactile temporal discrimination have been reported in writer's cramp patients [32]. However, it remains unclear what aspects of timing are affected by dystonia. Especially considering that timing is a multifaceted capacity that ranges from purely perceptual discrimination abilities to sensorimotor synchronization [33].

The extent to which dystonia is task-specific is a matter of debate. On the one hand, writer's cramp and MD patients have been found to show impairments in fine motor control tasks other than instrumental playing [11]–[13], [34], [35]. Furthermore, differences in brain activations have been found without the occurrence of dystonic movements [21]. On the other hand, MD is mainly seen as a task-specific disorder that impairs instrumental playing severely [1]. The disturbed temporal accuracy found in MD-patients during piano playing is most likely due to dystonic movements and not due to timing errors in temporal processing [9]. Furthermore, significant different activations between writer's cramp patients and healthy controls in writing with a pencil have been shown; whereas no difference between the patients and controls during writing with their finger were found [36]. This finding shows that dystonic symptoms may only be evoked during particular tasks.

The foregoing raises two mutually exclusive hypotheses. Firstly, if MD is also characterized by basic timing problems, then these problems should also occur when we test patients' timing abilities away from the instrument. Alternatively, if MD patients' impairments are mostly related to instrumental playing, then their basic timing perception and production capacities should be intact. The current study employs a battery of auditory-motor tasks to investigate basic timing-abilities of MD patients away from their instrument. If MD patients do not show impaired behavior on these tasks, this would support the task-specific nature of MD.

To test these hypotheses, we employed battery of auditory-motor tasks focusing on basic perceptual and action aspects of timing relevant for music making. The battery aims to separate purely perceptual timing capacities from timing production. Although the tasks included in the battery are not standard in clinical practice, all of them have been successfully employed in basic research on individual differences in perceptual and action aspects of sensorimotor timing in musicians [37]–[42].

Sensorimotor synchronization is the temporal coordination of an action with events in a predictable external rhythm. This fundamental human skill contributes to successful motor control in daily life and is important for musicians, because it plays an important role during ensemble music production. Precise and flexible sensorimotor synchronization requires mechanisms that enable an individual to adapt to timing variations and to anticipate tempo changes [43]–[45]. These underlying mechanisms were assessed in our task battery.

Furthermore, we used machine learning techniques to investigate whether MD patients are characterized by non-linear combinations of the timing abilities assessed in the battery. It has been suggested previously that instrument-specific performance differences between dystonia and control participants exist in particular combinations of timing variables instead of individual variables [10]. We extend this result to non-instrument-specific timing variables, investigating whether (non-linear) combinations of the timing variables measured here would identify patients and controls. To this end, we tested various supervised machine learning approaches in order to ascertain whether we could recognize patients by a signature consisting of various timing ability scores. If patients show the hypothesized timing problems, the present battery of tests would enable us to pinpoint at what stage of auditory-motor processing the deficit occurs. Furthermore, the machine learning approaches should be able to differentiate between MD patients and healthy controls based on the hypothesized pathological behaviors. If, on the contrary, no differences between MD patients and the matched control group are found, this would be in favor of the view that MD is mostly a task-specific motor impairment.

## Methods

### Participants

Fifteen patients (age 36.47±12.01 yrs., four females) with musicians' dystonia participated in the study. Patients were recruited from the outpatient clinic of the Institute of Music Physiology and Musicians' Medicine at the Hannover University of Music, Drama, and Media between February and June 2013. All patients were professional musicians between 18 and 65 years old. Inclusion criteria were right-handed patients with focal hand dystonia and isolated curling of the thumb, middle, ring or little finger when playing their instruments. Excluded were patients with embouchure dystonia, additional neurological problems or patients in which the right index finger was affected. All patients were diagnosed by a neurologist (author E.A.) specialised in movement disorders of musicians. For those patients who, prior to the experiment, received a botulinum toxin treatment (n = 6), the last injection was two to 24 months ago (8.6±8.3 months). This amount of time suggests that the effect of the injection had worn off by time the experiment took place. A description of the patients can be found in Table 1. The control group of 15 professional musicians without musicians' dystonia (age 36.13±12.59 yrs., five females) were matched to the patients as closely as possible for age, gender, handedness and musical instrument (Table 1).

**Table 1:** Description of the participants.

Participant	Gender	Age	Main instrument	Cumulative practice time (×10^3^ hours)	Experience (years)	Affected finger*		Months affected	Severity Score**	Self-rated playing ability***
p1	Male	40	Clarinet	13.10	30	4	Right hand	238	60%	80%
p2	Male	40	Clarinet	33.60	31	3	Left hand	150	90%	75%
p3	Female	23	Violin	20.20	18	4	Left hand	24	70%	60%
p4	Male	29	Piano	33.42	24	1	Right hand	27	70%	10%
p5	Male	19	Flute	9.04	12	4	Left hand	20	80%	50%
p6	Male	31	Guitar	26.21	20	3	Left hand	47	70%	65%
p7	Male	26	Cembalo	20.08	20	4	Right hand	20	80%	50%
p8	Male	43	Piano	24.20	30	3	Left hand	211	80%	80%
p9	Male	35	Guitar	23.10	22	4	Left hand	15	60%	20%
p10	Male	31	Piano	21.37	15	3	Right hand	19	90%	65%
p11	Male	55	Flute	78.35	45	4	Left hand	110	90%	70%
p12	Male	51	Guitar	50.04	39	4	Left hand	58	80%	70%
p13	Female	58	Violin	81.18	54	4	Left hand	125	90%	80%
p14	Female	52	Piano	51.50	38	4	Left hand	15	70%	30%
p15	Female	23	Guitar	5.57	13	3	Right hand	24	60%	25%
c1	Male	44	Clarinet	20.98	37					
c2	Male	42	Clarinet	9.33	32					
c3	Female	21	Violin	11.51	15					
c4	Male	30	Piano	30.43	23					
c5	Female	20	Flute	5.75	13					
c6	Male	29	Guitar	8.58	20					
c7	Male	25	Piano	11.28	20					
c8	Male	40	Piano	61.45	33					
c9	Male	35	Guitar	20.82	25					
c10	Male	31	Piano	14.70	26					
c11	Male	56	Flute	36.34	44					
c12	Male	55	Guitar	70.86	45					
c13	Female	54	Violin	20.64	46					
c14	Female	38	Piano	67.62	34					
c15	Female	22	Guitar	8.76	13					

^*^ 1 =  Thumb, 3 =  Middle finger, 4 =  Ring finger.

^**^ The severity score is based on expert rating: 100% =  healthy, 0% playing impossible.

^***^ Self-rated playing ability is judged by the patient self: 100% =  level before onset dystonia.

According to the laterality score from the Edinburgh Handedness Inventory all participants except one control participant (−100, fully left handed) were right handed (patients: 75.33±13.98/controls: 79.90±18.77). The study was approved by the local ethics committee of the Hannover Medical University. Following the Declaration of Helsinki, experimental procedures were explained to all participants and written informed consent was obtained prior to participation in the experiment. Control participants received a compensatory fee for their participation in the study.

### Tasks, procedures & measures

A battery of five auditory-motor tasks was employed to investigate at what stage of auditory-motor processing deficits occurs. The battery includes sensorimotor synchronization and perceptual tasks. The tasks were presented to the participants in a randomized order. Participants received oral and written instructions before each task. After the experiment, participants filled in a short questionnaire. In total the experiment took about 1–1.5 hours.

### Sensorimotor synchronization tasks: Adaptive tapping

**Figure 1:**
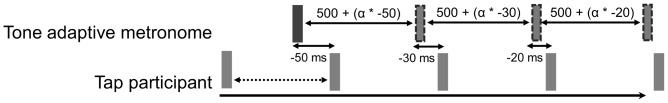
Pacing signal for the adaptive tapping task. The timing of the pacing signal was determined by the following equation: t_n+1_ =  t_n_ + 500 + α × asyn_n_. In the current experiment α was set to 0, 0.3 or 0.7, thus the pacing signal corrected 0 (non-responsive metronome in fixed trials), 30 or 70% of the asynchrony by shifting the next tone in the opposite direction.

The adaptive tapping task contained both, fixed and adaptive trials [46]. During the fixed trials participants synchronized their taps with a non-responsive metronome. During the adaptive trials the sequences responded to the participants' tap timing by implementing error correction [45]–[46]. In brief, the pacing sequence adjusted its timing during each trial based on the registered asynchrony between its previous tone and the participants' tap (phase correction). Two levels of error correction (α) were employed: 0.3 and 0.7. Each value indicates the proportion of each asynchrony that is corrected for by local adjustments to the timing of pacing events (Figure 1), resulting in an adaptive pacing sequence with which the participants synchronized their taps. The two levels of α were chosen to result in a hypothesized helpful (α = 0.3) metronome, that has previously been shown to boost sensorimotor synchronization and a hypothesized unhelpful (α = 0.7) metronome, leading to a more challenging synchronization task [46]–[47]. The non-responsive metronome in the fixed trials could be referred to as an adaptive metronome of which alpha is set to 0.

Stimulus presentation and tap recording was controlled by a MaxMSP program running PC with Windows. Participants tapped with their right index finger on a custom built tapping device, which was connected to PC and MaxMSP via a MIDI-connection. Stimulus sounds, sampled as a woodblock sound, were generated by a Roland SPD-S sampling pad. Sounds were presented over headphones and participants' taps did not trigger sounds.

The different conditions of the stable tapping task (α = 0 [fixed], α = 0.3, and α = 0.7) were presented in a randomized block of 10 trials. All trials had a base inter-onset interval of 500 ms and consisted of 42 tones. Participants were instructed to start tapping from the third tone onwards and to synchronize their taps as accurately as possible with the pacing signal, while maintaining the initial tempo. Prior to the experimental blocks participants performed one trial for each of the three conditions to familiarize themselves with the experimental procedure.

As a measure of sensorimotor synchronization accuracy the mean signed asynchrony between the metronome's tones and the participants' taps was calculated. The standard deviation of the signed asynchrony functioned as a measure of sensorimotor synchronization precision, indicating how consistent the taps were in relation to the tones [48]. The standard deviation of the signed asynchrony is also used as a measure of coupling-strength [46]–[47]. A lower standard deviation of the signed asynchrony reflects a stronger coupling between the pacing signal's tones and the participant's taps. Based on the stable tapping task, the amount of phase correction implemented by the participant was estimated as a measure of adaptation during sensorimotor synchronization [39]. The amount of human alpha can be determined based on the lag 1 autocorrelation of asynchronies in the conditions of stable tapping (α = 0 [fixed], α = 0.3, and α = 0.7). Based on a regression line, the alpha corresponding to a lag-1 autocorrelation of 0 was determined. To obtain an estimate of the amount of phase correction implemented by the participant this alpha value was subtracted from the hypothesized optimal amount of error correction, namely 0.9 [46], [49].

### Sensorimotor synchronization tasks: Tempo changing tapping

During the tempo changing tapping task participants were instructed to tap in synchrony with the tempo changing stimulus sequence. Twelve tempo changing sequences were employed [38]. The stimulus sequences consisted of 68 tones, starting with five tones with an inter-onset interval of 600 ms followed by tempo changes of which the inter-onset interval varied between 600 and 387 ms inter-onset interval. Tempo changes proceeded over the course of five to nine intervals, resulting in 12 slightly different sequences with nevertheless a similar character. All sequences contained eight continuous tempo changes resembling those found in performed music (i.e., accelerando and ritardando). Stimulus presentation and tap recording were controlled in the same way as during the stable tapping task. The order of the 12 sequences was randomized across participant. Participants performed two randomly chosen sequences as practice and to familiarize themselves with the experimental procedure.

The absolute mean asynchrony and the standard deviation of the signed asynchrony were calculated as measures of sensorimotor synchronization accuracy and precision, respectively [e.g., 38]. The cross correlations between the inter-stimulus and inter-tap intervals at lag 0 and lag 1 and the prediction/tracking ratio (PT-ratio) were calculated as indicators of anticipation mechanisms during sensorimotor synchronization. The PT-ratio is computed by dividing the lag 0 by the lag 1 cross correlation between inter-tap and inter-stimulus interval [38], [50]. If this ratio is greater than 1, it reflects the participant's tendency to predict the tempo change, while a ratio smaller than 1 indicates the participants tend to copy (track) the tempo changes. The PT-ratio has been shown to classify individual differences reliably and has been found to correlate positively with musical experience, tapping abilities and neural activation in different brain networks [38], [40], [50].

### Perceptual tasks: Beat Alignment Test

The Beat Alignment Test was an adapted version of the Beat Alignment Test developed by Iversen & Patel [37]. Since our participants were professional musicians, the adjustments were made to make the task more challenging (see Materials S1 for details). We chose five extracts (10–20 sec each) of musical recordings of various styles from Iversen and Patel's stimuli. After five seconds, a metronome was superimposed on the music. In half of the trials, the metronome was aligned with the beat of the musical piece. In the other trials, the metronome was phase-shifted to be either too late or too early by 10 or 15% of the average metronome click interval. A total of 40 stimuli (20 aligned, 20 misaligned) were randomly presented in four blocks of 10 trials. In between blocks, participants could take a short break. Participants were instructed to judge if the metronome was aligned with the beat of the musical piece or not.

The stimuli were generated offline and saved as wave files. A python-pygame graphical interface presented the instructions and stimuli and collected key press responses. Stimuli were presented through headphones. This task was used as a purely perceptual task to probe patients' capacity to align a metronome with the beat of a musical extract independently of their motor capacities [37]. Therefore, participants were explicitly instructed not to move or tap along while they were listening.

Prior to the experimental blocks, participants were presented an example with aligned metronome and one during which the metronome was shifted, i.e. misaligned trial. Next, participants completed a training block with four training trails (two aligned and two in which the metronome was shifted; +15% and −15% of the metronome interval). Participants responded whether the metronome was aligned or not. During the training block, but not during the experimental blocks, participants received accuracy feedback.

The summed correct responses divided by the total number of responses across extracts for each metronome shift (−15, −10, 0, +10, +15%) was calculated as an accuracy score for each participant.

### Perceptual tasks: Keystroke-sound delay detection task

At each trial, the participant pressed the “zero” key on the keypad at a time of her/his choosing and heard a tone. This tone was either played at the same time of the keystroke or delayed by a number of milliseconds [42]. The Maximum Likelihood Procedure (MLP) algorithm [51]–[52] was used to detect the threshold for the detection of the asynchrony between movement (keystroke) and the tone. The algorithm is designed to adaptively select the stimulus level (tone delay) on each trial so as to converge to the participant's threshold. For each block, the algorithm outputs an estimate for the participant's threshold. In short, the applied MLP algorithm works as follows: A set of candidate psychometric curves are maintained in parallel and for each, the likelihood of the set of the participants' responses is calculated. The psychometric curve that makes the participant's responses maximally likely is used to determine the stimulus level (the delay between the keystroke and the sound) on the next trial. We used 600 candidate psychometric curves with midpoints linearly spread between 0 and 600 ms delay, each combined with the five false alarm rates (0, 10, 20, 30, and 40%). Hence, a total of 3000 candidate psychometric curves were used. The source code for the MLP is freely available online on https://github.com/florisvanvugt/PythonMLP.

A USB keypad (Hama Slimline Keypad SK110) interfaced through HDI protocols with a python script was used to detect the keystroke onset and playing a woodblock wave sound (duration: 63 ms) through headphones.

Three experimental blocks were administered. These blocks consisted of 36 trials and contained six catch trials. Catch trials are trials on which the delay was set to 0 ms (regardless of the delay that was suggested by the MLP algorithm). The function of catch trials is to prevent participants from always responding “delayed” (which would cause the MLP algorithm to converge to a zero threshold). Catch trials were inserted randomly with the following constraints: the first 12 trials of each block contained 2 catch trials and the next 24 trials contained 4 catch trials.

Prior to the experimental blocks, participants first performed four training trials (two with no delay and two with a delay of 600 ms) to make clear the difference between when the sound came immediately and when it was delayed. During these practice trails participants received accuracy feedback about the given answers. Next, they performed a training block of 10 trials, starting at 600 ms delay but then using MLP to determine the stimulus levels of the following trials.

This task measured participants' sensitivity to asynchronies between motor (keystroke) and auditory (tone) events [42].

### Perceptual tasks: Anisochrony detection

Participants heard a five-tone sequence over headphones. The base sequence consisted of five isochronous sine wave tones (100 ms duration) presented with an IOI of 350 ms. In some trials, the fourth tone was delayed by a certain amount but the fifth tone was always on time [53]–[54]. That is, when the tone was delayed by an amount *d*, the third interval was longer by *d* ms and the fourth interval was shorter by *d* msec. The amount of delay depended on the participant's threshold, which was established adaptively using the MLP. The basic procedure was the same as for the delay detection task but for this task 200 logistic psychophysical curves were used. Midpoints of these curves were linearly spread over the 0 to 200 ms delay range (0% to 57% of the inter-tone intervals) and combined with the five false alarm rates (0, 10, 20, 30, and 40%). A python-pygame graphical interface presented the instructions and stimuli and collected keystroke responses.

Three experimental blocks of 36 trials, including six catch trials, were presented to the participants.

Participants were instructed to judge if the five-tone sequence was regular or irregular. Prior to the experimental blocks, four example stimuli (two regular, two irregular) were presented. For these trials participant received accuracy feedback. Next, a training block with 10 trials was administered.

The obtained threshold was used as an estimator of the precision of participants' auditory temporal perception [42], [53]–[54].

### Machine learning

#### Non-linear classification of patients and controls

In order to investigate whether the patient group was characterized by particular non-linear combinations of scores on the variables measured in this study, we performed supervised machine learning analyses as follows [for a similar procedure, see 10]. The variables that were fed into these analyses are outcome measures of the five tasks. From the stable tapping task we used the following variables. The mean signed asynchronies (ms) and the standard deviation of the asynchronies (ms) on the different levels of alpha (α = 0 [fixed], α = 0.3, and α = 0.7) were used, amounting to 6 data points (2 variables for each of the 3 levels of alpha) per participant. Furthermore, the error correction estimate (unit-less; see stable tapping methods) was used. From the tempo changing tapping task the mean absolute asynchrony (ms), the standard deviation of the signed asynchronies (ms), and the PT-ratio (unit-less) were included. For the perceptual tasks the score on the beat alignment test (%), the delay detection threshold (ms), and the anisochrony threshold (% of IOI) were fed into the supervised machine learning analyses.

Prior to running the machine learning analyses, each of the variables was rescaled and centred so that their mean was zero and standard deviation equal to one. Three established machine learning algorithms were tested (details below): Naive Bayesian classification, Linear Discriminant Analysis (LDA) and Support Vector Machines (SVM). Each model was given the variables as predictors and is trained to categorise participants as patient or control.

We first trained the models on the entire dataset, then removed the labels, and asked the model to predict which participants are patients and which are controls. We expected the models to do very well on this classification task, because the models have a large number of degrees of freedom. The risk of this great number of degrees of freedom is that we could over-fit the data, essentially fitting noise. In order to assess the models without risking over-fitting, we used leave-one-out-cross-validation (LOOCV). In this procedure, we trained each of the three models on all the data (training data) except one participant. The model is then tested on classifying this one participant (the test data). By repeating this procedure for each participant in the sample, we get an overall classification accuracy which is corrected for over-fitting. We then tested its overall success rate using binomial testing.

To perform naive Bayesian classification, the naiveBayes function from the e1071 machine learning package as part of the R package for statistical computing (version 3.0.2) was used. This function implements the standard Bayes classifier. To perform LDA, we used the lda function from the MASS package as part of the R package for statistical computing (version 3.0.2). Finally, SVM was implemented using the svm function from the e1071 machine learning package as part of the R package for statistical computing. We performed C-classification using a radial basis SVM kernel. Hyperparameter's cost and gamma were set to 10000 and 1e-4, respectively. These hyperparameter values were chosen from a range of possible values as those minimising the classification error. The machine used 22 support vectors.

### Data-analyses

The tapping data were processed with MATLAB (The Mathworks Inc, MA, USA R2011a). In addition to descriptive statistics, we performed mixed design ANOVAs (see below). If the assumption of sphericity was violated, the Greenhouse–Geisser correction was applied. To interpret the significant effects of the ANOVAs, the generalized η^2^ effect size was used. The effect sizes were interpreted according to Cohen's recommendation of 0.02 for a small effect, .13 for a medium effect, and .26 for a large effect [55]. The analyses were performed with SPSS (IBM SPSS Statistics 21) and R package for Statistical Computing (version 2.15.1).

## Results

Across the different tasks, no differences between left and right hand affected patients were observed. Therefore, in the analyses reported below all fifteen patients were included as a single level of the group factor.

### Sensorimotor synchronization tasks

#### Adaptive tapping

Two-way mixed design ANOVAs with group (patient or control) as between-participant factor, and level of alpha (three levels: α = 0 [fixed], α = 0.3, and α = 0.7) as within-participant factor were run to investigate differences between groups and the effect of the adaptive metronome on the mean and standard deviation of the signed asynchrony. In order to investigate whether the estimated amount of human phase correction differed between groups, an ANOVA with group (patient or control) as between-participant factor was run.

**Figure 2:**
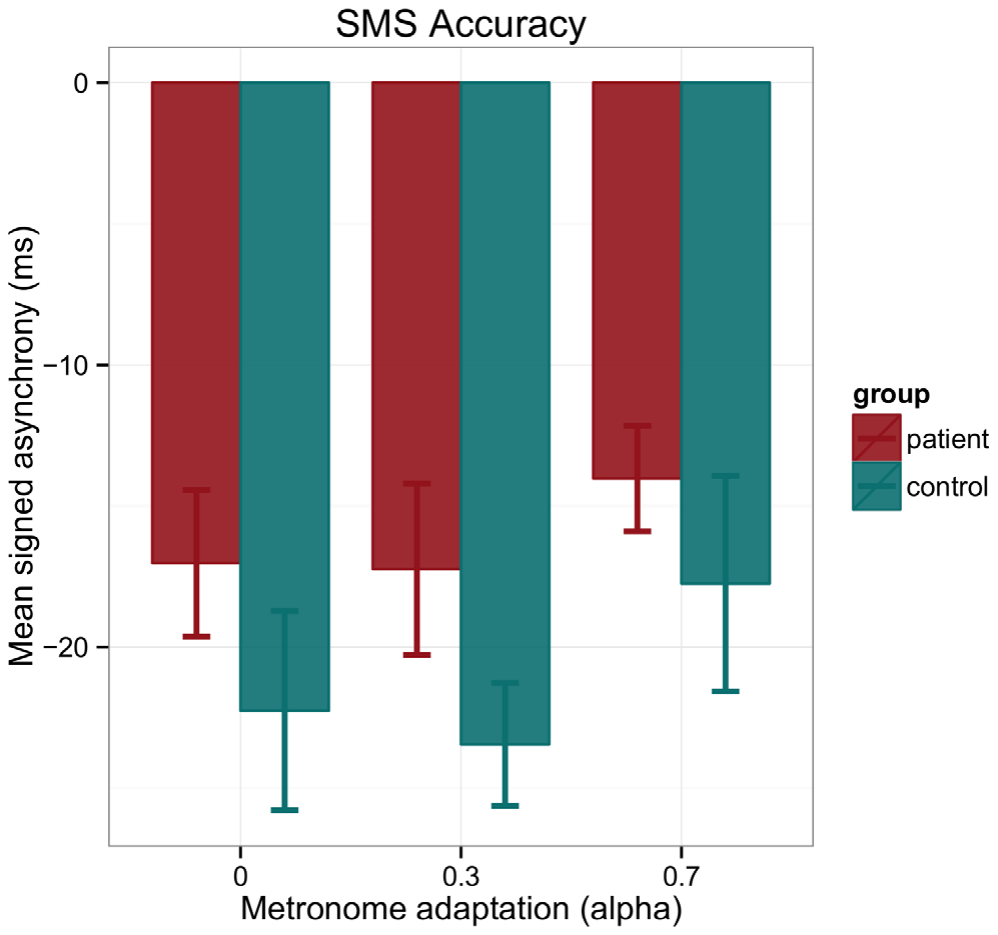
Adaptive tapping task accuracy results. Mean signed asynchronies as a measure of sensorimotor synchronization accuracy separated for group and levels of alpha. By convention negative values indicate that the tap preceded the tone. Error bars indicate standard error of the mean.

For the mean signed asynchrony there was no main effect of group [F(1,28) = 1.76, p = 0.20]. One control participant had unusual positive mean asynchrony, which was further than 2.5 SD away from the sample mean, for two of the three levels of alpha. When the analysis was repeated without this outlier a moderate significant main effect of group was found [F(1,27) = 4.61, p = 0.04, η^2^ = 0.15], indicating that the patients were more accurate in synchronizing their taps with the pacing sequence. In the analysis without the outlier, a moderate main effect of alpha was also found [F(1.63;44.05) = 9.31, p = 0.001, η^2^ = 0.25]. Pairwise comparisons revealed that synchronization was more accurate when the metronome implemented 70% phase correction compared to the fixed metronome (p<0.001) and the metronome that implemented 30% phase correction (p = 0.029). No significant interaction effect between group and alpha was found [F(1.63;44.05) = 0.41, p = 0.66] (Figure 2).

For the standard deviation of the signed asynchrony no significant main effects of group [F(1.28) = 0.10, p = 0.75] or alpha [F(2,56) = 2.75, p = 0.07], nor interaction effects [F(2,56) = 1.11, p = 0.33] were found. This indicates that the precision of synchronization did not differ between groups or levels of alpha (Table 2).

**Table 2:** Mean (SD) of the non-significant results for the different tasks and outcome measure separated per group and if applicable level of alpha.

Task	Measure	Patient group	Control group
Adaptive tapping task	Precision SD signed asyn (ms) alpha = 0 [fixed]	15.93 (3.05)	16.59 (4.45)
	Precision SD signed asyn (ms) alpha = 0.3	15.36 (2.58)	14.90 (3.23)
	Precision SD signed asyn (ms) alpha = 0.7	15.26 (2.03)	16.15 (4.65)
	Error correction estimate	0.61 (0.15)	0.53 (0.13)
Tempo changing tapping task	Accuracy mean abs asyn (ms)	36.47 (5.83)	37.90 (8.38)
	Precision SD sign asyn (ms)	36.67 (4.61)	36.50 (6.58)
	PT-ratio	1.04 (0.04)	1.05 (0.03)
Keystroke-sound delay detection task	Keystroke-sound delay detection threshold (ms)	86.8 (43.7)	104.3 (79.6)
Anisochrony detection task	Anisochrony threshold (% of inter-tone-interval)	4.5 (2.2)	4.7 (2.7)

There was also no significant difference in the estimated amount of error correction implemented by the patients and controls [F(1;28) = 2.60, p = 0.12] (Table 2).

#### Tempo changing tapping

Group differences between patients and controls for the absolute mean asynchrony, the standard deviation of the signed asynchrony and PT-ratio were statistically investigated by means of separate ANOVAs with the mentioned outcome measures as dependent variable and as between-factor group (patient or control). For all three measures no significant main effect of group was found (mean asynchrony [F(1;28) = 0.30, p = 0.59], standard deviation of asynchronies [F(1;28) = 0.01, p = 0.93]; PT-ratio [F(1;28) = 0.94, p = 0.34]) (Table 2). Furthermore, PT-indices estimated based on an autoregressive method [56] did not show a significant group difference [F(1;28) = 0.80, p = 0.38]. The correlation between the PT-index and PT-ratio was ρ = 0.97, p<0.001. These findings indicate that patients' synchronization abilities did not differ from controls for tempo changing sensorimotor synchronization accuracy and precision. Furthermore, both groups predicted the tempo changes to a similar degree (Table 2).

### Perceptual tasks

#### Beat Alignment Test

**Figure 3:**
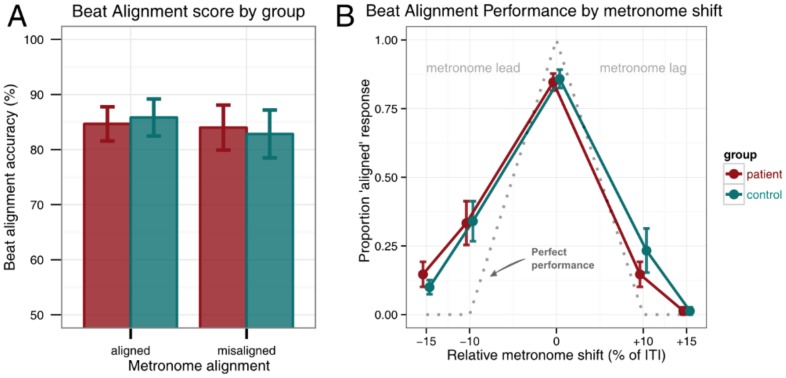
Beat alignment test results. (A) Overall accuracy scores. Error bars indicate standard error of the mean. (B) Aligned responses according to relative metronome shifts. Error bars indicate standard error of the mean.

In order to investigate whether beat alignment performance was different between groups, a mixed design ANOVA with between-participant factor group (patient or control) and within-participant factor metronome alignment (aligned or misaligned) was performed. The two groups had identical overall accuracy scores (84.3%). Therefore the main effect of group was not significant [F(1,28) = 0.00, p = 1.00]. The main effect of metronome alignment was not significant [F(1,28) = 0.17, p = 0.68], which indicated that participants were equally good at detecting aligned and misaligned metronomes. The interaction between metronome alignment and group was also not significant [F(1,28) = 0.07, p = 0.79] (Figure 3A).

In order to test whether the different metronome shifts differentially influenced performance, we proceeded to analyze the misaligned stimuli as follows. A mixed design ANOVA with within-factors shift direction (metronome lead or metronome lag), shift amount (10% or 15% of the inter-beat-interval) and between-factors group (patient or control) was performed. The dependent variable was the proportion “aligned” responses. The main effect of group was not significant [F(1,28) = 0.04, p = 0.85]. Shift direction revealed a small significant main effect [F(1,28) = 22.02, p<0.0001, η^2^ = 0.09], which indicated that participants responded “aligned” more often (erroneously) when the metronome preceded the beat (M = 23%, SD = 21%) than when the metronome came after the beat (M = 10%, SD = 14%). That is, participants more readily detected a metronome as misaligned when it came late than when it came early relative to the underlying musical beat. There was a moderate main effect of shift magnitude [F(1,28) = 30.86, p<0.001, η^2^ = 0.19], which revealed that participants judged the metronome aligned less often when it was shifted by 15% of the inter-beat-interval (M = 7%, SD = 8% “aligned” responses) than when it was shifted by 10% (M = 26%, SD = 25% “aligned” responses). The interaction between shift magnitude and direction was not significant [F(1,28) = 1.17, p = 0.29]. The interactions between group and shift magnitude or shift direction were not significant and there was no significant three-way interaction [all interactions F(1,28)<1.34, p>0.26] (Figure 3B).

#### Keystroke-sound delay detection task

Blocks in which participants responded “delayed” to more than 30% of catch trials were classified as invalid and eliminated from the analysis. This was the case of 14.9% of all blocks. A further 4.2% of blocks were discarded because they had not properly converged on a threshold. The criterion for non-convergence was if threshold estimates varied more than 2 ms/trial over the last ten trials (based on previous datasets [42]). After discarding blocks, participants had on average 2.5 (SD = 0.73) valid blocks remaining. The average threshold (in ms) for these remaining blocks was calculated.

In order to investigate whether keystroke-sound delay detection differed between groups, we performed an ANOVA with delay detection threshold as dependent variable and between-factor group (patient or control). There was no effect of group [F(1,28) = 0.55, p = 0.46], which indicated that delay detection thresholds did not differ between patients and controls. One participant (control group) had an unusually high delay detection threshold (314.5 ms), which was further than 3 SD away from the mean of the sample. Repeating our analysis without this participant, we still found no effect of group [F(1,27) = 0.02, p = 0.90] on delay detection threshold (Table 2). The thresholds for both controls and patients were comparable to previously observed thresholds for delay detection in musician populations [42].

#### Anisochrony detection

Blocks in which participants responded “irregular” to more than 30% of catch trials were eliminated. This was the case in all three blocks of one participant (3.2% of all blocks). This control participant was eliminated from further analysis. All blocks had properly converged according to the non-convergence criterion (less than 1.18 ms/trial threshold change over the last ten trials). After discarding, all remaining participants had all 3 blocks remaining. The average threshold (in % of the inter-tone-interval) was calculated for these remaining blocks.

In order to investigate whether anisochrony detection differed between groups, an ANOVA with anisochrony threshold as dependent variable and between-factor group (patient or control) was performed. There was no significant main effect of group [F(1,27) = 0.07, p = 0.80] which revealed that anisochrony thresholds were identical for patients and controls (Table 2). The thresholds for both controls and patients were comparable to previously found thresholds for anisochrony in musician populations [42], [53].

### Machine Learning

#### Non-linear classification of patients and controls

One participant from the control group was not included in this analysis due to a lack of valid blocks for anisochrony threshold (see anisochrony results above).

All three methods of machine learning (Naive Bayes, LDA and SVM) were able to classify participants as patient or control above chance level (all binomial test p< = 0.001; Table 3) when they were trained on the entire dataset. However, when controlling for over-fitting using leave-one-out-cross validation (LOOCV), all models performed at chance level (all binomial test p>0.36; Table 3). This indicated that combinations of variable scores that separated patients and controls could be identified, but that these combinations were based on individual differences unrelated to MD.

**Table 3:** Classification accuracy for each of the machine learning approaches: Naive Bayesian, Linear Discriminant analysis (LDA) and Support Vector Machines (SVM).

Method	Accuracy	Patient predictive value	Control predictive value	Sensitivity	Specificity	Binomial test p-value
SVM.all	86.2%	86.7%	85.7%	86.7%	85.7%	<0.001*
SVM.LOOCV	44.8%	47.1%	41.7%	53.3%	35.7%	0.771
LDA.all	82.8%	85.7%	80.0%	80.0%	85.7%	<0.001*
LDA.LOOCV	55.2%	55.6%	54.5%	66.7%	42.9%	0.356
NaiveBayes.all	79.3%	76.5%	83.3%	86.7%	71.4%	0.001*
NaiveBayes.LOOCV	51.7%	52.9%	50.0%	60.0%	42.9%	0.500

For each approach, the classification rate for the model that was trained on all data (all) and the model that was tested using leave-one-out-cross validation (LOOCV) is reported. We report accuracy (number correct divided by total number of participants), patient-predictive-value (the proportion of true patients among those classified as patients by the model), control-predictive-value (the proportion of true controls among those classified as controls by the model), sensitivity (the proportion of participants classified as patients relative to the total number of patients), specificity (the proportion of participants classified as controls relative to the total number of controls), binomial test p-value.

## Discussion

This study investigated MD patients' timing perception and production capacities away from their instrument. Previous studies showed that beside the task specific problems, MD patient are also impaired in more general tasks and processes [5], [12]. Results of the current study suggest that basic movement and timing capacities relevant for music making are unaffected in MD patients. Both for purely auditory perception tasks as well as for the sensorimotor synchronization tapping tasks, no impairments were found in MD-patients. Furthermore, state-of-the-art machine learning algorithms could not separate patients and controls based on the outcome measures of the five timing tasks. Overall, these results suggest that MD-patients show intact auditory-motor processing related to the basic timing tasks studied here. Therefore, the present results support the claim that MD is a task-specific movement disorder.

The finding that patients are more accurate in synchronizing their taps with the tones in the stable tapping task is most likely related to their amount of practice. Patients reported on average a higher amount of accumulated hours of practice [patients: 32.7 (23.0)/controls: 26.6 (22.4)×10^3^ hours], indicating that they practiced more than the tested controls. It has been shown that practice is associated with high sensorimotor synchronization accuracy [50], [57]. The positive effect of the adaptive timing of the pacing signal on sensorimotor synchronization was only visible for sensorimotor synchronization accuracy. The coupling between the pacing signal and the participants' taps was very high, indicated by the small asynchronies. This tight coupling is not very surprising considering we tested professional musicians but also did not leave much room for improvement in sensorimotor synchronization behavior. This might also be the reason why the hypothesized unhelpful metronome (alpha  = 0.7) did show a positive effect of sensorimotor synchronization accuracy but the hypothesized helpful metronome (alpha  = 0.3) did not reveal a difference compared to the unresponsive metronome (alpha  = 0 [fixed]). On the small asynchronies only a 70% correction led to a meaningful adjustment of the timing of the metronome's tones.

The Beat Alignment Test was used to measure participants' perceptual precision in detecting whether a metronome was aligned with a musical beat. Humans perceive a regular pulse of the rhythm of the musical pieces [58]. Not surprisingly the bigger (15%) metronome misalignments were easier to detect for participants than the more subtle 10% phase-shifted metronome. Furthermore, the finding that late, positive shifts are easier to detect than early, negative shifts might be explained by means of the oscillator-based dynamic theory of attending [59]. According to this theory, regular sequences establish internal oscillators that resonate in phase with the regular external stimulus. The attention of the listener is not equally distributed of the entire time span but follows attentional cycles that are linked to the internal oscillators. The perception (and production) of events is more accurate when the event coincides with the peak of the attentional cycle [59]–[60]. In case of the phase-shifted metronome the clicks do not coincide with the expected pulse of the music. In case of the late shifted metronome, the narrowed focus around anticipated events may increase attention as time progresses, because the expected click has not yet occurred [59], [61]. Furthermore, in trials where the metronome was shifted earlier the internal oscillator is disturbed by one cue. This cue is the result of the pulse of the too early occurring metronome click. Trials during which the metronome was shifted later two cues are available, namely the missing click when a click was expected and then the click that happens after the perceived pulse [62]. Late shifts might therefore be easier to detect and classify as misaligned, than early shifts [59],[62]. Surprisingly, Lim and colleagues [3] showed that if the last tone in a five tone sequences occurred earlier this was more easily detectable than if the fifth tone was delayed. Furthermore, they concluded that this difference was bigger in MD patients. The anisochrony task employed here, with an adaptively delayed tone, has strong resemblances with the task employed by Lim and colleagues [3]. However, the anisochrony task did not reveal this difference in detecting delayed tones between the MD patients and healthy controls. The effect found by Lim and colleagues [3] might arise because musicians perhaps link delays at the end of the five tone sequence to the final tone of a musical phrase. In expressively timed musical performances this final tone is often delayed. It was previously found that detecting delays at the end of a musical phrase is difficult, because musicians expect delays at this point [63]–[64]. In the anisochrony task the one-but-last tone was delayed instead of the last tone, this phrase-final lengthening effect did not occur. Here, we aimed to purely measure participants' auditory temporal perception and there we eliminated potential interference from high-level musical processing by measuring sensitivity to the fourth (i.e. one-but-last) instead of fifth (last) tone.

In summary, the observed significant effects are all related to the applied experimental manipulations but did not differ between patients and controls. Furthermore, even state-of-the-art machine learning algorithms were not able to pick up pathological behaviors that would tease apart patients and controls. The results suggest that basic timing abilities, both perception and production, are intact in patients that suffer from MD. Although this finding supports the claim that MD is a task-specific movement disorder our main hypothesis was that, due to the assumed maladaptive plasticity in brain areas that are highly relevant for timing, patients would show impaired timing abilities compared to a matched healthy control group.

A possible explanation for the lack of the effect of MD on timing might be related to the nature of the task. The tasks employed in the current study are less complex than the movements involved in music making. The tasks were specifically developed and successfully employed in previous studies addressing basic perceptual and action aspects of timing that are important for playing music [37]–[42]. Although differences in brain activations have been shown by very basic tasks that did not evoke dystonic symptoms (e.g., finger tapping), the functional maladaptive plasticity underlying focal hand dystonia was more pronounced in more complex tasks (e.g., Luria apposition task) [21], [26]. It would therefore be interesting to test MD-patients' timing abilities using more complex tasks.

A second explanation might be found in the role of the affected finger. In the current experiment, focussing on basic timing abilities, participants of whom the right index finger was affected were excluded from the study. The reason to do this was to be sure that no dystonic movements would be evoked during the tasks, which often required participants to use their right index finger. Dystonic movements most likely would disturb the temporal accuracy. Previous studies reporting timing anomalies in MD-patients during piano playing [9] and individuated finger movements [10] indicated the role of the affected finger(s). Jabusch and colleagues [9] found no difference in timing parameters between the unaffected hands of the MD patients and the reference hands of the healthy controls. Obviously, in the affected hands of the tested pianists, dystonic cramping was present during the task. The results by Furuya and Altenmüller [10] were also obtained by means of a finger-tapping task. But in this case participants were instructed to depress the piano keys with all fingers, while tapping with one of the fingers. This made the task more complex, but more importantly also included the affected finger in the position of the hand, like during real piano playing. Furthermore, patients were directly tested at their instrument (like in [9]).

The latter two points brought up in relation to the Furuya and Altenmüller [10] study link to a final and most likely reason why no general basic timing deficits were found in MD-patients, namely the task-specific nature of this movement disorder. Hu and colleagues (2006) found differences in brain activation in writer's cramp patients compared to healthy controls only for writing with a pencil. However, no differences between patients and controls were found for the same writing task when performed with the finger [36]. This finding pin-points the specific role of the task in task-specific hand dystonia. The problems patients suffering from MD encounter are most strongly related to instrumental playing [1]. However, a recent study found that 98% of the patients also report problems with other fine motor control daily life activities, such as computer keyboard typing and hand writing [13]. Similarly, in our sample 9 out of 15 patients reported (subtle) problems with non-musical fine motor tasks. But the loss of voluntary motor control in MD-patients is most pronounced in the over-practiced task, for these professional musicians playing their instrument. The timing abilities of MD-patients were tested away from their instrument, since we were interested in the basic aspect of timing. The question remains if problems with timing are present in MD-patients when basic features of timing are investigated at the instrument without evoking dystonic movements that disturb temporal aspects of the movement.

Furthermore, we did not measure brain activation patterns during the tasks. Therefore, it remains unclear whether the previously found maladaptive plasticity [20]–[21] played a role during the experiment. Moreover, a recent study found that focal hand dystonia patients exhibited decreased activations and increased connectivity in different brain regions (e.g., cerebellum, putamen, and sensorimotor cortex). Nevertheless, identical motor performance in the patient and the healthy control group was found, suggesting that differences in activation and connectivity may reflect beneficial compensatory processes [65]. The tasks employed in the current experiment focus on basic perceptual and action aspects of timing that have been found to be important for playing music and ensemble music making [42], [48]. Due to the importance of movement timing for musicians, it might be that MD patients have developed compensatory mechanisms to maintain their extraordinary level of timing. We addressed this issue by examining the amount of phase correction and the PT-ratio, as indicators of the underlying mechanisms of successful sensorimotor synchronization [45]. The finding that these measures also did not differ between groups speaks against the use of compensatory processes by patients, but further research is necessary to definitively exclude this possibility.

The timing of movements is obviously a complex multifaceted capacity that entails both perceptual and action components, also seems to differ between types of movements (e.g., continuous vs discrete tasks). Although the employed battery of auditory-motor tasks covers a wide range of processes relevant to motor timing, it is impossible to test all aspects exhaustively. The aspects that we investigated are mostly relevant to discrete movement tasks, such as finger tapping. It is therefore unknown whether our findings generalize to timing abilities most relevant in other tasks, such as emergent timing in continuous movements [66]–[67].

Further experiments could further clarify the task-specific nature of MD and the generalizability of our results to other types of timing tasks. In these experiments the above-mentioned points could be tested. For example, sensorimotor synchronization abilities of right index finger affected pianists could be addressed via a similar paradigm both at the piano as well as using the current experiment set up. Also more complex tasks, such as tapping tasks that include multiple fingers or even both hands, and continuous tasks could be administrated while timing measures are recorded. The tasks could be administrated in an fMRI scanner to reveal the brain activation patterns of patients and controls during the tasks.

Overall, results of the current study suggest that basic timing abilities stay intact in patients that suffer from MD. This finding supports the idea that MD is a task-specific movement disorder and that problems in this patient population are most pronounced in relation to instrumental playing. The current study raises the question how patients' basic timing capacities can be intact although they are impaired at a variety of fine motor tasks. Our results suggest that MD patients may maintain their musical timing skills by practicing sensorimotor timing tasks away from their instrument. Also, the finding that these basic musical skills are intact might suggest that if musical instruments are adapted in such a way as to not evoke dystonic movements, musical performance in these patients may be restored.

## Supporting Information

Materials S1 (DOCX)
