# Supplementary material for: Basic Timing Abilities Stay Intact in Patients with Musician's Dystonia
Source: PLoS One. 2014 Mar 25;9(3):e92906. doi: 10.1371/journal.pone.0092906 (PMC3965486; doi:10.1371/journal.pone.0092906)
Supplement: Materials S1 — (DOCX) [file pone.0092906.s001.docx]

**Beat Alignment Task (BAT)**

*Materials*

The following 5 extracts were selected from among the stimuli used by Iversen & Patel (2008). We indicate the mean inter-onset-interval of the metronome clicks and their standard deviation. The coefficient-of-variation indicates the metronome standard deviation divided by the mean inter-onset-interval.

| **ID** | **Style** | **Piece** | **Artist** | **Mean IOI (ms)*** | **SD IOI (ms)** | **CV IOI (%)** |
| --- | --- | --- | --- | --- | --- | --- |
| HTH | Rock | Hard to handle | Black Crowes | 560.3 | 11.6 | 2.07% |
| HSG | Rock | Hurts so good | J. Mellencamp | 478.0 | 12.1 | 2.53% |
| KPS | Jazz | King Porter stomp | Glenn Miller | 696.3 | 13.8 | 1.98% |
| ACL | Pop Orchestral | A chorus line | Boston Pops | 485.8 | 13.8 | 2.84% |
| SMA | Pop Orchestral | Superman | Boston Pops | 735.2 | 56.0 | 7.62% |

*Metronome*

Our metronome sound was a 1 kHz sine wave with 50msec duration, instead of 100msec as in Iversen & Patel (2008). Furthermore, we used only a phase-shifted metronome and omitted Iversen & Patel's condition with a period-shifted metronome.

*Instructions*

The instructions are adapted from the BAASTA (Farrugia et al., ICMPC 2012). However, the instructions were changed in the following ways. Firstly, Farrugia et al. asked their participants whether the metronome was "regular" or not, whereas we asked whether it was aligned or not. Secondly, Farrugia et al.'s instructions defined rhythm by referring to how one could tap along, but later ask people not to tap along.

1. Iversen JR, Patel AD (2008) The Beat Alignment Test (BAT): Surveying beat processing abilities in the general population. In: Miyazaki K, Hiraga Y, Adachi M, Nakajima Y, Tsuzaki M, editors. Proceedings of the 10th International Conference on Music Perception & Cognition (ICMPC10) Sapporo, Japan. Adelaide: Causal Productions. pp. 465-468.
2. Farrugia N, Benoit CE, Harding E, Kotz SA, DallaBella, S (2012) BAASTA: battery for the assessment of auditory sensorimotor and timing abilities. In: Cambouropoulos E, Tsougras P, Mavromatis P, Pastiadis K, editors. Proceedings of the Joint Conference: 12^th^ Biennial International Conference on Music Perception and Cognition and 8^th^ Triennial Conference of the European Society for the Cognitive Sciences of Music (ICMPC-ESCOM2012) Thessaloniki, Greece. pp. 292-299.
